# Wnt5a-mediated autophagy promotes radiation resistance of nasopharyngeal carcinoma

**DOI:** 10.7150/jca.71526

**Published:** 2022-04-24

**Authors:** Zhaoyi Lu, Yandan Zhou, Qiancheng Jing

**Affiliations:** 1 The Affiliated Changsha Central Hospital, Department of Otolaryngology Head and Neck Surgery, Hengyang Medical School, University of South China. Changsha, Hunan, 410001, People's Republic of China.; 2 Changsha Aier Eye Hospital, Aier Eye Hospital Group, Changsha, Hunan,410000, People's Republic of China.; 3 Department of Otolaryngology Head and Neck Surgery, Xiangya Hospital, Central South University, 87 Xiangya Road, Changsha, Hunan 410008, People's Republic of China.

**Keywords:** Wnt5a, autophagy, radiation resistance, nasopharyngeal carcinoma, Head and Neck Squamous Cell Carcinoma

## Abstract

Wnt signaling pathways and autophagy play an essential role in tumor progression. Canonical Wnt signaling pathways in radiation resistance have been studied in the past, but it remains unclear whether the noncanonical Wnt signaling pathways can affect tumor radiation resistance through protective autophagy. Nasopharyngeal carcinoma, a particular subtype of head and neck squamous cell carcinoma, relies on radiation therapy. In this study, we found that radioactive rays could significantly promote the expression of Wnt noncanonical signaling pathways ligands in nasopharyngeal carcinoma, among which Wnt5A was the most markedly altered. We have demonstrated that Wnt5a can reduce the radiation sensitivity of nasopharyngeal carcinoma *in vitro* and *in vitro* experiments. Meanwhile, we found much more greater autophagosomes in overexpressed-Wnt5A nasopharyngeal carcinoma cells by electron microscopy. Further mechanism exploration revealed that Beclin1 is the main target of Wnt5A, and knocking down Beclin1 can partially reduce Wnt5a-induced radiation resistance. By studying Wnt5A-mediated protective autophagy in promoting radiation resistance in nasopharyngeal carcinoma cells, we hope that the Wnt5A and Beclin1 can become effective targets for overcoming radiation resistance in the future.

## Introduction

Head and neck squamous cell carcinoma (HNSCC) is the seventh-largest tumor globally 1 and is mainly associated with heavy tobacco and alcohol intake 2. In recent years, with the decrease in tobacco consumption, the proportion of patients with tumors increased by HPV infection (mainly by HPV 16) 3, 4. Nasopharyngeal carcinoma differs from other subtypes of head and neck squamous cell carcinoma in epidemiology, pathology, natural history, and treatment. Patients with nasopharyngeal carcinoma are mainly concentrated in southeast Asia. EBV virus infection is often relevant to pathologically confirmed non-keratinized squamous cell carcinoma (more than 95% of endemic areas).

Radiation therapy plays a significant role in the radical and comprehensive treatment of head and neck squamous cell carcinoma. Nasopharyngeal carcinoma is sensitive to radiation therapy, the most crucial treatment method in premetastatic tumors 5. Although with the progress of radiotherapy technology, the curative effect is guaranteed and the side effects are gradually reduced, about 50% of high-risk patients still have a local recurrence within three years of follow-up after radiotherapy 6. Finding the cause of radiation resistance is always the focus of modern radiotherapy research.

The Wnt signaling pathways are divided into canonical and noncanonical signaling pathways. The non-canonical signaling pathways include the β‐catenin-independent WNT signaling pathways, planar cell polarity (PCP) signaling pathways and the Wnt/Ca+ signaling pathways 7. WNT5A, WNT5B, and WNT11 are the most representative noncanonical Wnt signaling pathways ligands among the 19 WNT genes, mediated by acting on FZD3 or FZD6 receptors, and ROR1, ROR2, or PTK7 co-receptors. Wnt5a is a noncanonical ligand with high conservatism of evolution, mainly plays a role in cell development, such as polarity, proliferation, differentiation, adhesion, and migration 8. Abnormal activation or inhibition of Wnt5a is an important marker of tumor progression 9, 10, play the role of antitumor effect in thyroid cancer 11 and breast cancer 12. Still, it promotes cancer in melanoma 13, adenocarcinoma 14, colorectal cancer 15, leukemia 16, small cell lung cancer 17 and stomach cancer 18.

In addition to acting on noncanonical Wnt signaling pathways such as PCP and the Wnt/Ca^2+^, Wnt5a can also function through canonical Wnt signaling pathways under certain conditions 19. Studies suggest that Wnt5a's role in tumors may be related to pathways for downstream action, where activation of CamKII induces anti-cancer effects. In contrast, activation of the PKC, β-catenin and PCP pathways promotes tumor progression 20. At present, it has been found that Wnt5a play an important role in tumor proliferation 16, 21, invasive metastasis 22, 23, metabolism 24, 25, inflammation 26, 27, aging 28, stemness 29, immunosuppressive tumor microenvironment 30 and chemotherapy resistance 31, 32. There is still a lack of research on the correlation between Wnt5a and radiation resistance, which deserves further investigation.

In 1963, Christian de Duve first proposed autophagy, and in 2016, Yoshinori Ohsumi won the Nobel Prize for his research on the mechanism of autophagy. Autophagy is ancient and highly conserved catabolism that can form two-membranous vesicles (autophagosomes), engulfing cellular proteins and organelles, transporting them to lysosomes, and allowing cells to quickly adapt to a stressful or hostile environment. Autophagy is controlled by highly regulated signaling events at various cellular states and is induced by different signals and cellular stress. It is often thought that autophagy can prevent tumor progression; increased autophagy flow can promote tumor survival and growth once the tumor is formed. Autophagy enhancers have been reported to prevent tumor progression 33 in precancerous lesions or early tumors. In contrast, both autophagy agonists and inhibitors can promote progression in reports of advanced cancer 34-36. Similarly, studies have shown that radiation can induce the onset of autophagy. Still, part of it promotes the sensitivity of radiation therapy, while the other part shows inhibition of radiation sensitivity 37. Some mechanisms of inhibition in autophagy sensitization radiotherapy have been attributed to the decrease in γ-H2AX formation and DNA damage repair 38.

Earlier, we conducted in-depth research on the canonical Wnt/β‐catenin signaling pathways. We found that Wnt3a, as a canonical ligand of the Wnt/β-catenin signaling pathways, can be used as a biomarker for head and neck squamous cell carcinoma 39 and can lead to radiation resistance by promoting protective autophagy 40. In the sequencing data 41, we simultaneously observed a significant upregulation of Wnt5A, Wnt5B, and Wnt11 in the Wnt noncanonical signaling pathways, and qPCR verified the change. By mining the TCGA-HNSC (the Cancer Genome Atlas- Head and Neck Squamous Cell Carcinoma) database, although none of the three ligands had prognostic value, we found that Wnt5a was significantly upregulated in head and neck squamous cell carcinoma (Fig S1). Although Wnt5a has been associated with tumor progression and autophagy, the study of radiation therapy resistance of Wnt5a in head and neck squamous cell carcinoma is unclear. There are no related studies on whether Wnt5a can affect radiation resistance by influencing autophagy. This article will focus on the mechanism of a Wnt noncanonical signaling pathways ligand-Wnt5a in radiation resistance to head and neck tumors.

## Materials and methods

### Cell culture

Nasopharyngeal carcinoma cell lines 6-10B, and CNE2 were purchased from the Cell Center of Central South University (Changsha, China). The media were supplemented with 10% fetal bovine serum and 1% penicillin-streptomycin, cultured in a 37 °C 5% CO_2_ incubator, and both were passaged and experimented with at the exponential phase.

### Transfection

To overexpress Wnt5a, we infect cells with lentivirus (EX-Z0683-Lv201, GeneCopoeiaTM), stable cells are screened with puromycin for two weeks, and the efficiency of gene regulation is assessed by Western blotting. An empty vector was used as a control. For Beclin1 knockdown, three siRNA were used, and target sequences were as follows: siRNA 1:5′‐GGA GAT CTT AGA GCA AAT GAA‐3′, siRNA 2:5′‐GGA CAC GAG TTT CAA GAT CCT‐3′, siRNA 3:5′‐CCA ACG TCT TTA ATG CAA CCT‐3′. The siRNA 1, which is the most efficient, was used to design an shRNA and package lentiviral vector pLKO.1 (Addgene).

### Irradiation

The linear accelerator (2100EX, Varian Medical Systems, Palo Alto, CA, USA) offers a 6‐MeV electron beam of 300 cGy/min. A 1.5 cm compensation gel is covered in a cell culture vessel 100 cm from the radioactive source.

### Plate clonogenic survival assays

After cells are irradiated with a gradient radiation dose, their radiation resistance is assessed by clonal survival. 6-well plates are inoculated with 600 cells per well, and different plates correspond to different radiation doses. After irradiation, the cells were cultured for 14 days, and the clonal survival rate (a colony with > 50 cells) was counted, as before 41. Repeat three times per experiment.

### Immunofluorescence microscopy analysis

We spread crawlers (12 mm) in a 24-well plate to seed cells. After six hours of incubation under 4Gy irradiation and non-irradiation control, the foci of γ-H2AX were compared. Statistical method for foci of γ-H2AX refers to the previous report 42.

### Xenograft tumor model

Athymic male nude mice were purchased from Hunan SJA Laboratory Animal Co., Ltd. (Changsha, Hunan, China). Animal Ethics Committee of Central South University (Changsha, Hunan, China). Inoculate the right hip back at 5-7 weeks, respectively (cells overexpressing Wnt5a and control), 2 million cells each. To be grown to 100 mm^3^, receive two doses of radiation for a total of 8 Gy. The control group was made sham‐irradiated. Then, we measured the mice's body weight and basic condition during the experiment. Tumor volume measured every 2-3 days (volume = length × width^2^/2). All rats were executed two weekends after the first radiation. All xenograft tumors were dissected, and weight was measured for statistical analysis.

### Bioinformatics and Statistical analysis

mRNA expression data from TCGA-HNSC (TCGA, Firehose Legacy, https://www.cbioportal.org/), immunohistochemistry from The Human Protein Atlas (https://www.proteinatlas.org/). The data were made using the mean ± SD, and a student's t-test was performed to calculate the statistical significance of differences between groups using GraphPad Prism. Survival curves were used by the Kaplan Meier method, and analyzed by the log-rank test. Correlation of gene expression (Wnt5a and Beclin1) was assessed using Pearson Correlation Coefficient. P‐value < 0.05 in a two‐tailed test makes sense.

## Results

### Wnt5a contributes to radiation resistance of nasopharyngeal carcinoma

**Figure 1:**
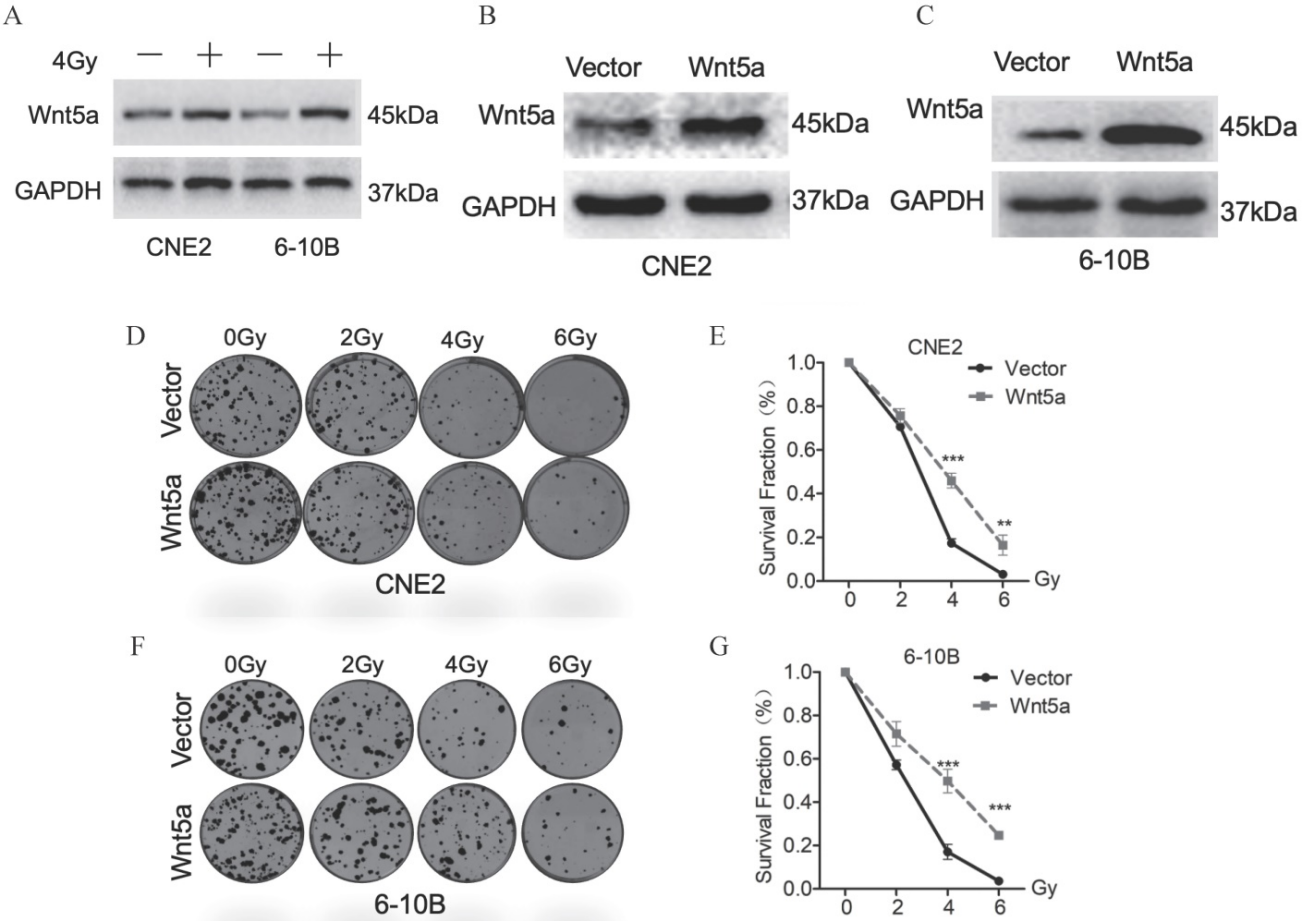
** Wnt5a contributes to radiation resistance of nasopharyngeal carcinoma. (A)** Western blotting assays were used to evaluate the expression of proteins in the Wnt5a in CNE-2 and 6-10B cells exposed to 4 Gy irradiation. **(B-C)** Nasopharyngeal carcinoma CNE2 and 6-10B cells were infected with lentivirus mediated Wnt5a or control cDNA and then subjected to puromycin screening for 2 weeks. Western blotting was used to examine the expression of Wnt5a protein in CNE2 and 6-10B cells. **(D & F)** Cells overexpressing Wnt5a or control cDNA were irradiated with the indicated doses of irradiation and clonogenic assays were obtained after 2 weeks. **(E & G)** Survival curves of cells in each group were determined by clonogenic assays. **p < 0.01; ***p < 0.001.

Based on our previous research 41, we further explore the role of the Wnt noncanonical pathway ligand-Wnt5a in irradiation. We exposed CNE2 and 6-10B to 4Gy irradiation and extracted proteins 24 hours later. Western blotting assays detection showed that Wnt5A proteins were significantly upregulated in CNE2 and 6-10B cell lines (Fig. 1A). These results indicate that irradiation activates the Wnt5a expression in nasopharyngeal carcinoma cells. To explore the effect of Wnt5A on radiation sensitivity in nasopharyngeal carcinoma cell lines (6-10B and CNE-2), we constructed stable cell lines with overexpression of Wnt5A. We then verified its expression efficiency using Western blotting assays (Fig. 1B, C). Through gradient dose irradiation (2, 4, 6Gy), we observed in the plate cloning experiment that the Wnt5a overexpressed strain showed more cell survival at 4 and 6Gy irradiation dose (Fig. 1D, F). Statistical analysis of the plate colony verified our observed results (Fig. 1E, G).

### Exogenous rhWnt5a promotes radiation resistance to nasopharyngeal carcinoma

**Figure 2:**
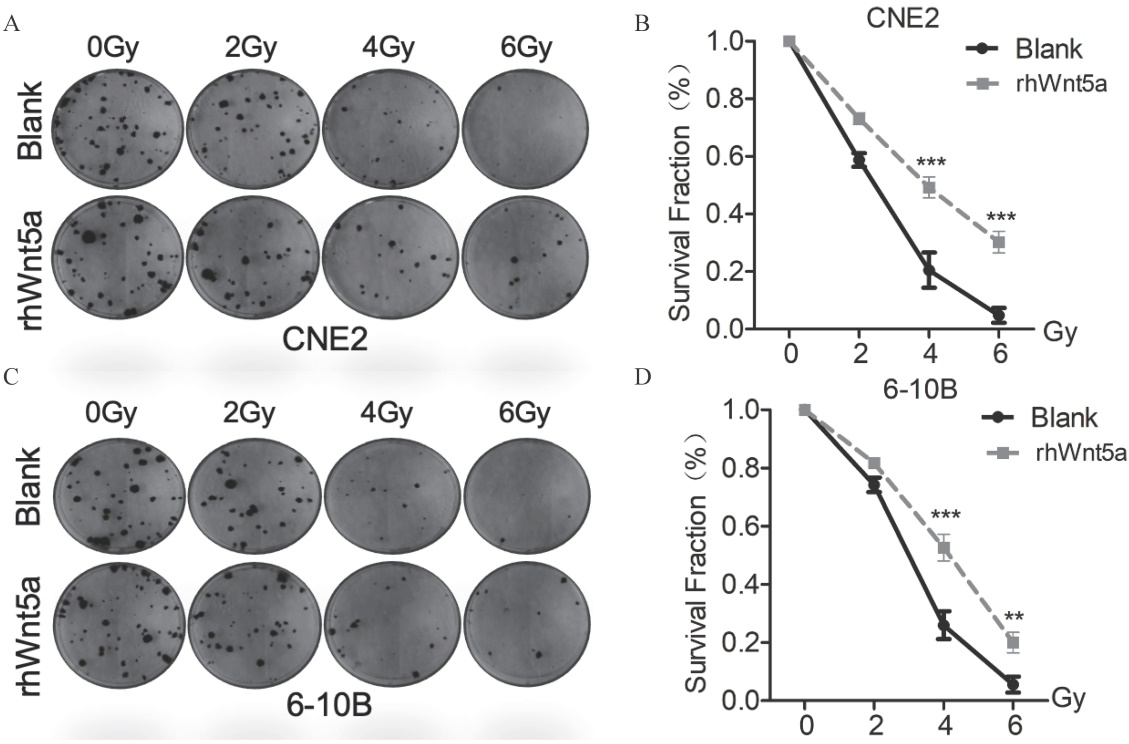
** Exogenous rh-Wnt5a promotes radiation resistance to nasopharyngeal carcinoma. (A & C)** Cells with medium added exogenous rh-Wnt5a and solvent were irradiated with the indicated doses of irradiation and clonogenic assays were obtained after 2 weeks. **(B & D)** Survival curves of cells in each group were determined by clonogenic assays. **p < 0.01; ***p < 0.001.

To verify the role of WNT5a protein in nasopharyngeal carcinoma radiation resistance, we supplemented exogenous WNT5a protein. By plate cloning experiment (Fig. 2A, C), we observed similar results with overexpression of WNT5a. Both CNE2 and 6-10B with exogenous supplementation of WNT5a protein showed increased cell viability at radiation doses with 4 and 6Gy (Fig. 2B, D), which may indicate that WNT5a protein promotes radiation resistance of nasopharyngeal carcinoma *in vitro*.

### Wnt5a inhibits the levels of DNA damage

**Figure 3:**
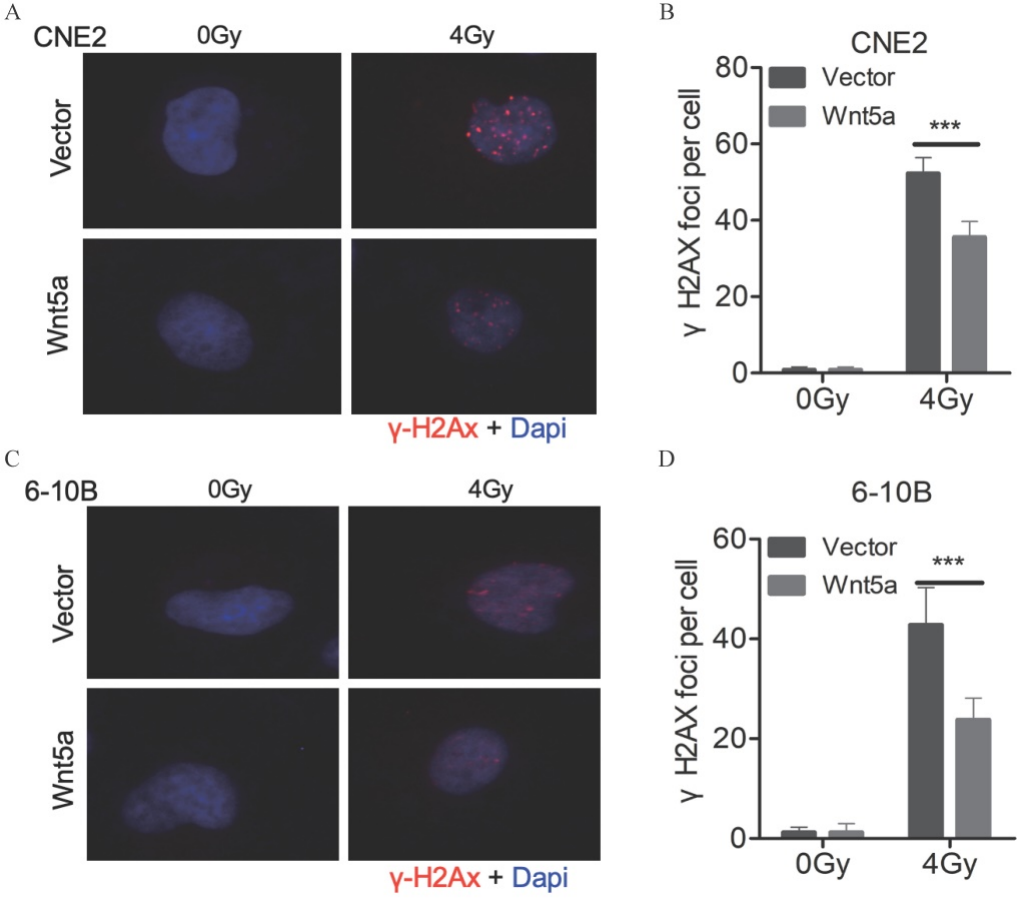
** Wnt5a inhibits the levels of DNA damage. (A & C)** Immunofluorescence staining was used to confirm the γ- H2AX foci in CNE2 and 6‐10B cells at 6 hours post‐irradiation with 4 Gy irradiation. **(B & D)** γH2AX foci were quantified by immunofluorescence staining. ***p < 0.001.

The levels of DNA damage can be assessed with the number of γ-H2AX foci (Fig. 3A, C). We found that under 4Gy radiation dose, the number of γ-H2AX foci decreased in CNE2 and 6-10B with overexpressed WNT5a (Fig. 3B, D). It may indicate that overexpression of WNT5a may inhibit levels of DNA damage.

### Wnt5a enhances radiation resistance *in vivo*

**Figure 4:**
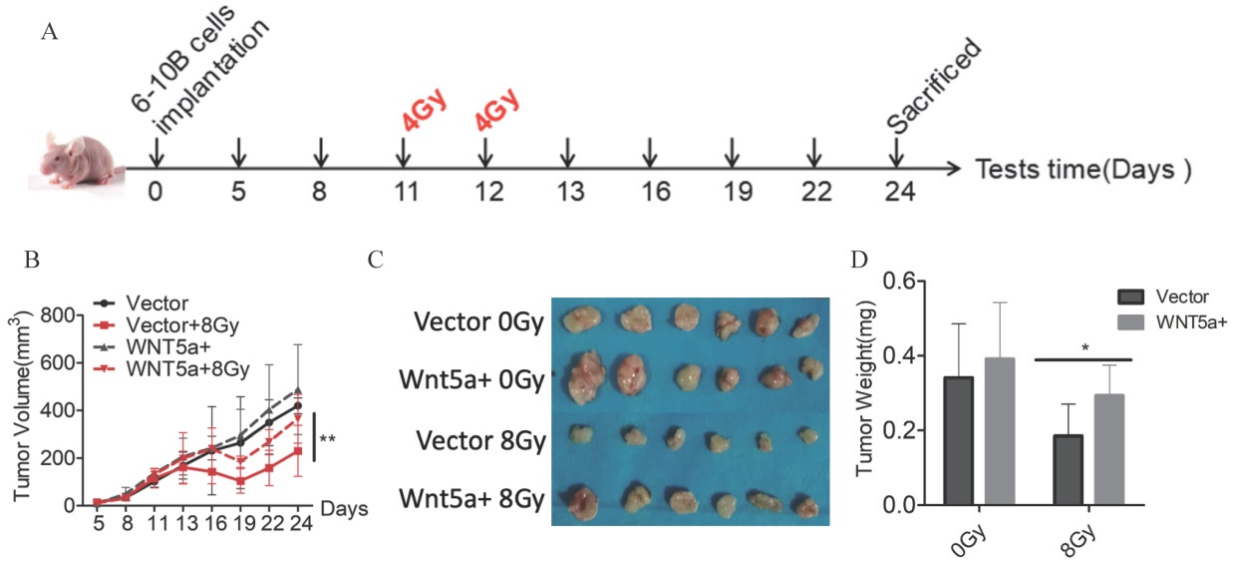
** Wnt5a enhances radiation resistance *in vivo*. (A)** Schematic flow of the experimental design. Nude mice were subcutaneously injected with 6‐10B cells overexpressing Wnt5a and then subjected to two consecutive 4 Gy irradiation treatments treatment when the tumor volume reached 100 mm^3^. Mice were finally killed at day 23 after tumor injection. **(B)** Tumor volumes were measured with callipers every 3‐4 days to monitor the growth pattern of mice. **(C)** The final gross tumors were captured. **(D)** Average tumor weight in each group was compared. *p < 0.05; **p < 0.01.

To further verify our findings, we constructed a nude-mouse transplanted tumor model and administered 4Gy of irradiation on the 11^th^ and 12^th^ day after planting 6-10B cells with control and Wnt5a-overexpression (Fig. 4A). In the groups that did not receive irradiation, there was no difference in tumor growth between the control and over-expressed WNT5a groups (Fig. 4B, C). In contrast, in the irradiation group, the over-expressed WNT5a tumor grew faster and showed resistance to radiation. Meanwhile, we found by weighing the tumor that the overexpressed WNT5a tumors were heavier in the irradiation group, while there was no significant difference in the non-irradiation group (Fig. 4D). Taken together, our data indicate that overexpression of Wnt5a inhibits the therapeutic efficiency of radiotherapy in HNSCC.

### Wnt5a enhances autophagy

**Figure 5:**
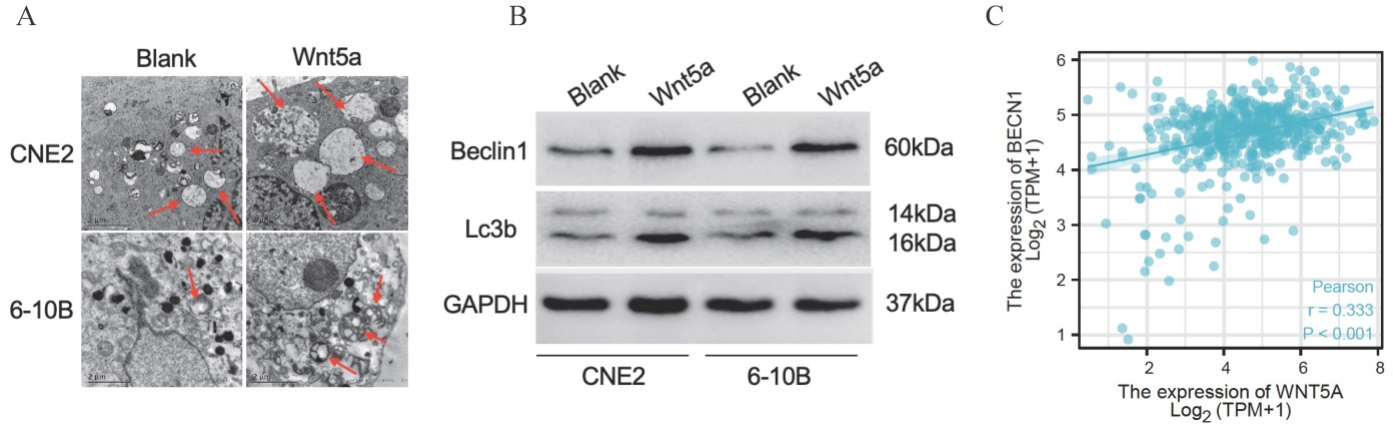
** Wnt5a enhances autophagy. HNSCC CNE2 and 6‐10B cells were infected with lentivirus mediated Wnt5a or control cDNA and then subjected to puromycin screening for 2 weeks. (A)** Representative images of autophagosomes (indicated by red arrows) observed by transmission electron microscopy in CNE2 and 6‐10B cells at 3 hours post 4 Gy irradiation exposure. **(B)** Western blotting was used to examine the expression of proteins associated with the Wnt5a and autophagic proteins (Beclin1 and LC3B). **(C)** The Pearson rank correlation coefficient validated the positive correlation between Wnt5a and Beclin1.

Literature reported that Wnt5A could activate the noncanonical WNT signaling pathways to promote autophagy 42-43. We found much greater autophagosomes in overexpressed-Wnt5A CNE2 and 6-10B cells by electron microscopy (Fig. 5A). Meanwhile, the protein Beclin1 and LC3B was significantly upregulated in Wnt5A-overexpressed CNE2 and 6-10B cells by western blotting assays, and the changes of autophagy flow indicated that Wnt5A might promote autophagy (Fig. 5B). Further analysis showed that mRNA expression level of Wnt5A was significantly positively correlated with Beclin1 in TCGA-HNSC data, which provided a direction for further mechanism research (Fig. 5C).

### Wnt5a enhances radiation resistance to nasopharyngeal carcinoma through autophagy

**Figure 6:**
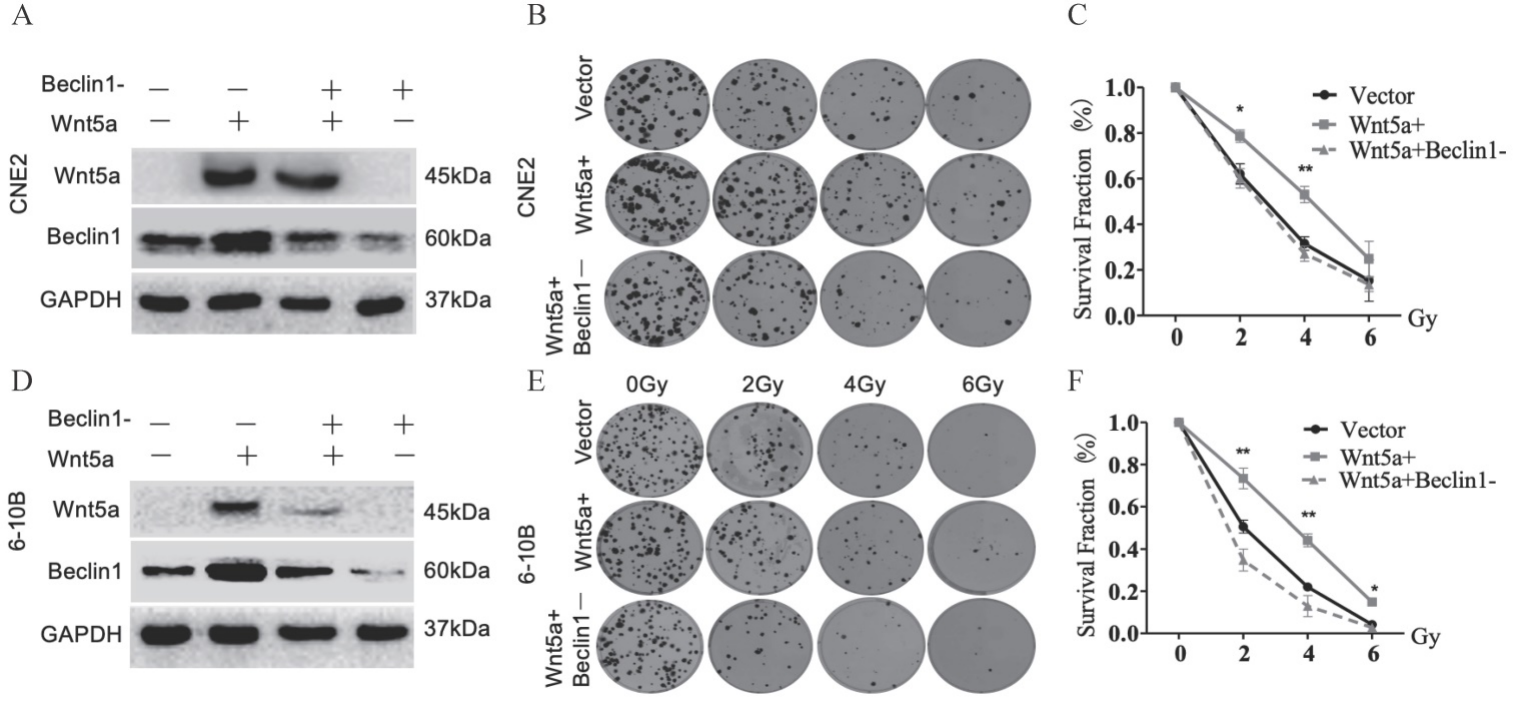
** Wnt5a enhances radiation resistance to nasopharyngeal carcinoma through autophagy.** HNSCC CNE2 and 6‐10B cells were infected witt lentivirus‐mediated Wnt5a or control cDNA and then infected with Beclin1 shRNA to inhibit its expression. **(A & D)** Western blotting assays were used to evaluate the transfection efficiency. **(B & E)** Cells in each group were irradiated with the indicated doses of irradiation and clonogenic assays were obtained after 2 weeks. **(C & F)** Survival curves of cells in each group were determined by clonogenic assays. *p < 0.05; **p < 0.01.

We confirmed the regulatory efficiency of Beclin1 shRNA in control and Wnt5a overexpressed nasopharyngeal carcinoma cell lines by WB (Fig. 6A, D). Plate cloning experiment in Wnt5a-overexpressed 6-10B cells with Beclin1 shRNA showed decreased viability at an irradiation dose of 2, 4, 6Gy (Fig. 6B-C). Similarly, Wnt5a-overexpressed CNE2 cells showed decreased viability after knocking down Beclin1 at an irradiation dose of 2, 4Gy (Fig. 6E-F). It may indicate that Wnt5A may promote radiation resistance through protective autophagy mediated by the key factor Beclin1.

## Discussion

Radiotherapy plays an important role in head and neck squamous cell carcinoma, especially nasopharyngeal cancer. Radiotherapy alone or systemic therapy is an important treatment method for locally recurrent head and neck squamous cell carcinoma. Radiotherapy combined with chemotherapy, targeted therapy and immune agonist is expected to make precise therapy possible, especially for patients with an HPV infection with better benefits.

Radiation resistance often occurs in patients with advanced head and neck squamous cell carcinoma, which has become the bottleneck of radiotherapy efficacy. It is essential to study the sensitization of biologically targeted therapy to radiotherapy 44. Radiation resistance can be divided into endogenous and acquired. The mechanism studies 45 have mainly found adaptive pathways (Cyclin D1, NF-κB, Survivin, ROS), DNA damage repair pathways (DNA-PK, RAD51, ATM), adhesion pathways (Integrins, FAK, Paxillin), inflammation pathways (TNF-α, IL, TGF-β, NF-κB, STAT3, Cox-2), developmental pathways (Hedgehog, Wnt/β-catenin, Notch), Hypoxic pathways (HIF-1, VEGF) and RTK-PI3K/Akt pathways (EGFR, STAT, MAPK, PI3K/Akt).

There are 19 Wnt signaling pathways ligands in the human genome, and Wnt5A was the most strongly expressed ligand in the tumor center in early studies of head and neck squamous cell carcinoma 46. Although Wnt5A plays a controversial role in tumor progression, its role in promoting the progression of head and neck squamous cell carcinoma is evident 29, 46. We found that the Wnt signaling pathways were significantly activated by sequencing radiation-resistant strains of nasopharyngeal cancer cell lines, and further studies suggested a strong correlation with autophagy 41. In this study, we further found that in addition to the canonical Wnt ligand (Wnt3a) in the Wnt ligand family can cause radiation resistance 40, the noncanonical ligand Wnt5a can also promote radiation resistance, but whether its function depends on β‐catenin is still worth further study. In the literature, Wnt5a has been reported to have the specificity of tumor activity (differentiation, proliferation, and invasion) 46 in head and neck squamous cell carcinoma. We found that Wnt5a was highly expressed in tumors relative to the para cancer, and the sequencing results of the resistance strain also showed higher expression of Wnt5a. It satisfies our precise strategic consideration of tumor radiation sensitization; through the inhibition of Wnt5a or its downstream target gene Beclin1, we can accurately reverse the radiation resistance effect of tumor cells without too many side effects on normal cells. At present, the research conclusions on the effect of autophagy on radiation sensitivity show significant differences. *In vitro* and *in vivo* studies on the radiation resistance model in nasopharyngeal carcinoma, we found that radiation can induce the overexpression of Wnt canonical signaling pathways (represented by Wnt3a) and Wnt non-canonical signaling pathways (represented by Wnt5A). Meanwhile, they all can mediate Beclin1, which improves autophagy levels and leads to radiation resistance. Still, it may have something to do with the environment the cells are in, the functions of the Wnt canonical signaling pathways and Wnt noncanonical signaling pathways in autophagy and radiation resistance are the independent effect is necessary to study further.

Autophagy plays a vital role in induction of tumor development, stemness maintenance and therapy resistance 47. In radiation-resistant cells, Bcl-2 is upregulated to inhibit apoptosis 48, while Bcl-2 is reported to inhibit Beclin1-dependent autophagy, thereby maintaining basal autophagy levels that contribute to cell survival 49,50. Our previous report showed that the Wnt3a-Beclin1 axis promotes radiation resistance in studies showing that tumors may utilize protective autophagy as a strategy for their survival 40. As an essential protein for regulating autophagy, Beclin1 has a dual role in tumors by removing defective and damaged organelles and other cellular components to inhibit tumorigenesis. It can also induce cell carcinogenesis and progression 51. In a randomized cohort study of 128 cases of nasopharyngeal carcinoma, Beclin1 was associated with a poor prognosis 52, 53, and was consistent with our prognostic analysis at HNSC-TCGA. Zhou et al. found that radiation therapy induces autophagy levels to improve the survival rate of nasopharyngeal carcinoma cells, while autophagy inhibitors chloroquine and 3-MA can promote cell death 54-57. Both autophagy and Wnt signaling pathways have essential links to cell development and homeostasis 58, most studies on autophagy have focused on Wnt/β‐catenin but ignored noncanonical Wnt signaling pathways. Our research further uses transmission electron microscopy to detect autophagosomes in Wnt5a-overexpressed nasopharyngeal carcinoma cells and controls, we found that autophagosomes significantly increased size and number in Wnt5a-overexpressed cells relative to controls. Beclin1 is a critical protein in the occurrence of autophagy, and its protein levels and transcriptional levels are significantly highly expressed in HNSCC patients (Fig. S2A-B). Overall survival and progress free survival assay revealed that a low expression of Beclin1 was correlated with better prognosis of HNSCC patients (Fig. S2C-D). After correlation analysis of gene expression in TCGA-HNSC data and verification in western blotting assays, we found that Wnt5a mainly regulated the expression of Beclin1 and mediated changes in autophagy levels and eventually led to radiation resistance. These analyses indicate that activation of the Wnt5a-Beclin1 pathway may contribute to the progression of HNSCC, even radiation resistance.

At present, many reports have confirmed the feasibility of targeted therapy sensitization radiotherapy 59. In this study, we found that WNT5A was an effective target for radiotherapy resistance, which combined with autophagy molecule provides a promising new therapeutic strategy for advanced HNSCC patients. However, there are still some shortcomings in the study. Next, we will explore whether WNT5A has the same effect of radiotherapy resistance in other HNSCC tumors, such as hypopharyngeal cancer. More effective sensitization targets still need to be explored and transformed through clinical trials as soon as possible.

## Supplementary Material

Supplementary figures.
