# Supplementary material for: Wnt5a-mediated autophagy promotes radiation resistance of nasopharyngeal carcinoma
Source: J Cancer. 2022 Apr 24;13(7):2388–96. doi: 10.7150/jca.71526 (PMC9066197; doi:10.7150/jca.71526)
Supplement: Supplementary file 1 — Supplementary figures. [file jcav13p2388s1.pdf]

Supplementary file:

Figure S1. Transcriptome-level and survival analysis of WNT5A in head and neck squamous cell carcinoma. (A-B) Transcriptional expression of WNT5A is significantly high in representative non-canonical Wnt signaling pathways ligands and HNSCC patients (both non-match data and matched data, mRNA expression data from TCGA-HNSC (TCGA, Firehose Legacy, https://www.cbioportal.org/), (C-D) Kaplan-Meier curve analysis of overall survival and progress free survival of TCGA-HNSC patients in high- and low-WNT5A expression groups($p > 0.05$).

Figure S2. Protein levels, transcriptome-level, and survival analysis of Beclin1 in head and neck squamous cell carcinoma. (A-B) Transcriptional expression and protein level of Beclin1 is significantly high in HNSCC patients (both non-match data and matched data, mRNA expression data from TCGA-HNSC (TCGA, Firehose Legacy, https://www.cbioportal.org/), Protein level from The Human Protein Atlas (https://www.proteinatlas.org/)). (C-D) Kaplan-Meier curve analysis of overall survival and progress free survival of TCGA-HNSC patients in high- and low-Beclin1 expression groups ($p < 0.05$)

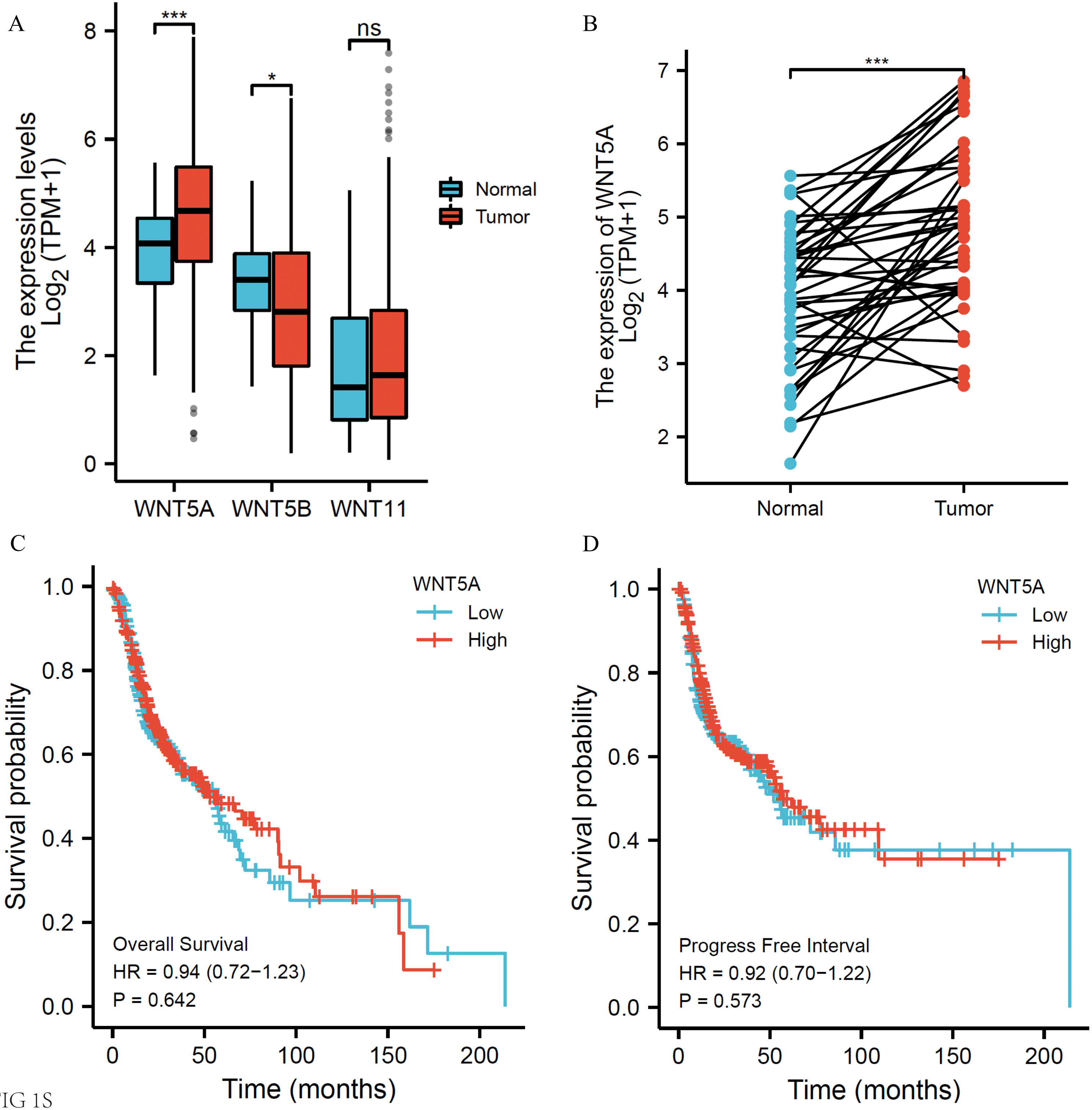
A
B
C
D
The expression levels
Log_2 (TPM+1)
***
*
ns
Normal
Tumor
WNT5A
WNT5B
WNT11
The expression of WNT5A
Log_2 (TPM+1)
Survival probability
Time (months)
WNT5A
Low
High
Overall Survival
HR = 0.94 (0.72−1.23)
P = 0.642
Progress Free Interval
HR = 0.92 (0.70−1.22)
P = 0.573

FIG 1S

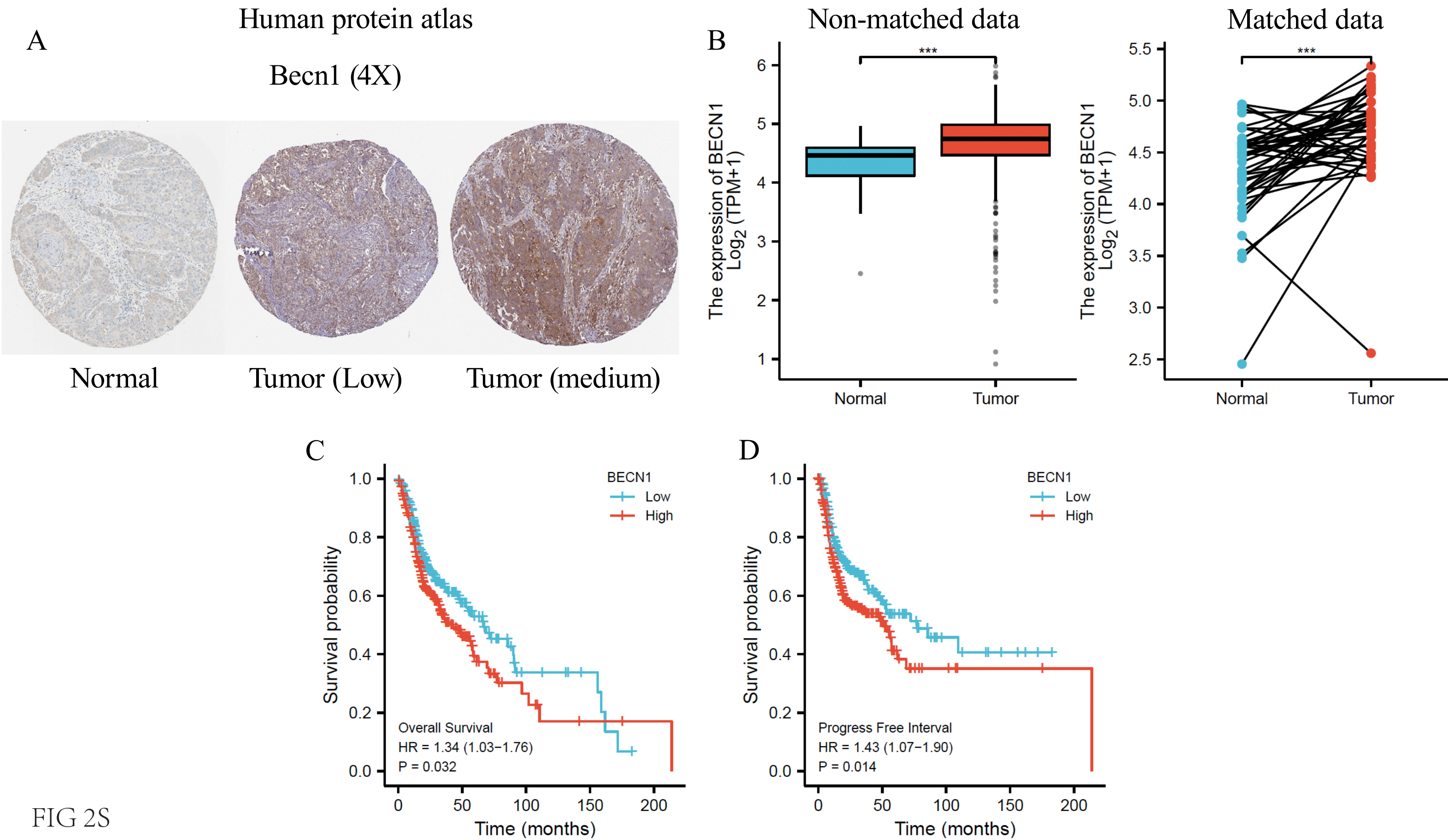
A
Human protein atlas
Becn1 (4X)
Normal
Tumor (Low)
Tumor (medium)
B
Non-matched data
Matched data
***
The expression of BECN1
Log2 (TPM+1)
Normal
Tumor
C
D
BECN1
Low
High
Survival probability
Time (months)
Overall Survival
HR = 1.34 (1.03−1.76)
P = 0.032
Progress Free Interval
HR = 1.43 (1.07−1.90)
P = 0.014

FIG 2S
